# Preparation and Properties of Mechanically Robust, Colorless, and Transparent Aramid Films

**DOI:** 10.3390/polym16050575

**Published:** 2024-02-20

**Authors:** Heesang Kim, Jin-Hee Noh, Young-Rae Kim, Hyojin Kim, Giseop Kwak

**Affiliations:** 1 Polymeric Nanomaterials Laboratory, Department of Polymer Science & Engineering, Kyungpook National University, 1370 Sankyuk-Dong, Buk-Ku, Daegu 41566, Republic of Korea; wsk1025@naver.com (H.K.); jhnoh@dgtp.or.kr (J.-H.N.); xornjs147@naver.com (Y.-R.K.); 2 Advanced Materials & Components Center, Industry Innovation Division, Daegu Technopark, 46-17 Seongseogongdan-ro, Dalseo-gu, Daegu 42716, Republic of Korea

**Keywords:** aramid film, acid removal, tensile strength, elastic modulus, transmittance, yellow index

## Abstract

In this study, various diamine monomers were used to synthesize aramid polymer films via a low-temperature solution condensation reaction with diacid chloride. For diamines with relatively high basicity, the reaction system became opaque because amine salt formation inhibited polymer synthesis. Meanwhile, low-basicity diamines with strong electron-withdrawing groups, such as CF_3_ and sulfone, were smoothly polymerized without amine salt formation to provide highly viscous solutions. The acid byproduct HCl generated during polymerization was removed by adding propylene oxide to the reaction vessel and converting the acid into highly volatile inert substances. The resulting solutions were used as varnishes without any additional purification, and polymer films with an excellent appearance were easily obtained through a conventional casting and convection drying process. The films neither tore nor broke when pulled or bent by hand; furthermore, even when heated up to 400 °C, they did not decompose or melt. Moreover, polymers prepared from 2,2-bis(trifluoromethyl)benzidine (TFMB) and bis(4-aminophenyl)sulfone (pAPS) did not exhibit glass transition until decomposition. The prepared polymer films showed a high elastic modulus of more than 4.1 GPa and a high tensile strength of more than 52 MPa. In particular, TFMB-, pAPS-, and 2,2-bis(4-aminophenyl)hexafluoropropane-based polymer films were colorless and transparent, with very high light transmittances of 95%, 96%, and 91%, respectively, at 420 nm and low yellow indexes of 2.4, 1.9, and 4.3, respectively.

## 1. Introduction

Aramids (aromatic polyamides), as well as polyimides and polyamideimides, are well-known excellent engineering polymers [1,2,3]. Poly (p-phenylene terephthalamide) (PPTA), commercially known as Kevlar, is a representative aramid polymer [1,4]. PPTA not only has high resistance to heat and chemicals but also has extremely high tensile strength, affording fibers stronger than steel [5]. Because aramid polymers have a rigid rod-like backbone based on a sp^2^ hybridized, coplanar structure comprising aromatic and amide groups, the conformational change that inevitably accompanies thermodynamic phase changes is extremely limited, resulting in slight entropy changes [6]. In addition, because hydrogen bonding between the polymer chains is greatly amplified by the amide groups, the internal cohesion in the solid state is significantly enhanced, thereby requiring an extremely high amount of enthalpy for phase change [7]. Therefore, aramids are widely used in materials that require high thermal resistance, thermodynamic dimensional stability, and mechanical robustness. Further, because aramids are highly (electro)chemically stable due to their resonance structure, they can be applied to materials that require insulation and chemical resistance. Despite these advantages, aramid polymers are infusible, and the production and processing of aramids into films is difficult due to their poor solubility in common organic solvents. Particularly, because conventional aramids are yellow, processing them into transparent films seems impossible. Moreover, owing to their intrinsically low hydrodynamic chain mobility, their molecular weight cannot be significantly increased during polymerization, and salt bridge gelation may occur.

Common manufacturing methods for aramid polymers include low-temperature solution condensation polymerization and interfacial polymerization using diamine and diacid chloride as monomers [1]. Interfacial polymerization requires additional processes, such as filtration, drying, and redissolution, after polymerization, which is unattractive because it increases manufacturing costs. Meanwhile, in solution condensation polymerization, because aprotic polar amide solvents such as N,N-dimethylacetamide (DMAc) and N-methyl-2-pyrrolidone are used to increase polymer solubility, relatively high molecular weight polymer films can be obtained. In addition, as these solvents serve as agents that trap HCl acid byproducts from a reaction system at a low temperature of approximately 0 °C, they suppress the formation of insoluble amine salts, allowing polymerization to proceed without diamine loss [8]. However, the formation of amine salts may significantly depend on the basicity of the diamine monomer used, and once formed, the amine salts may cause salt bridge gelation, thereby preventing smooth polymerization. Even if polymerization is achieved successfully, an additional purification process to remove the acid byproducts should be preceded to produce a film.

**Figure 1:**
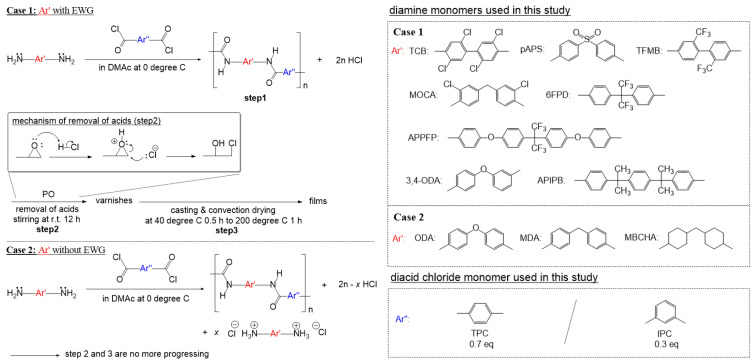
Synthetic route for aramids in this study.

Aiming at alleviating the above issues, we found an excellent method in U.S. patents that can easily and conveniently produce colorless and transparent aramid polymer films without any special purification process. Harris et al. [9,10] used 2,2-bis(trifluoromethyl) benzidine (TFMB) as a diamine monomer and reacted it with a diacid chloride mixture of terephthaloyl chloride (TPC) and isophthaloyl chloride (IPC) in DMAc to produce high-viscosity varnishes. Then, propylene oxide (PO) was added to the same reaction vessel to convert the HCl acid byproducts into inert, highly volatile substances, eliminating the need for additional purification (Case 1 in Figure 1). Notably, colorless and transparent films were obtained using the solution immediately after polymerization as a varnish. The polymer films were not only optically colorless and transparent but also had a low thermal expansion coefficient of less than 10 ppm/°C, ultimately resulting in excellent thermodynamic dimensional stability. Despite the convenience and ease of colorless and transparent aramid film production, applications of this manufacturing method to other aramid films have not yet been reported.

In this study, we applied Harris’ method to other diamine monomers and examined the thermal, mechanical, and optical properties of the produced films. As in TFMB, diamines with low basicity provided high-viscosity polymer solutions without amine salt formation. Subsequently, by adding PO to the reaction vessel, the HCl acid byproduct was easily removed, and the reaction solution was used as varnish without any additional purification to produce films with a good appearance (Case 1 in Figure 1). Meanwhile, diamine monomers with relatively high basicity lost their reactivity by forming amine salts during polymerization. Owing to salt formation, the reaction system became opaque, and polymers were not obtained (Case 2 in Figure 1). The varnishes manufactured from Case 1 had a high viscosity in a wide range depending on the monomer used, from which polymer films of appropriate thickness could be obtained. Through ^1^H nuclear magnetic resonance (^1^H-NMR) and Fourier-transform infrared (FTIR) spectroscopic analysis, we confirmed that the polymers were well-combined with aromatic and amide groups, as desired. The heat resistance as well as thermodynamic and mechanical excellence of the polymer films were confirmed through thermogravimetric analysis (TGA), differential scanning calorimetry (DSC), and universal testing machine (UTM) analyses. All polymers had very high decomposition temperatures (greater than 424 °C) and did not melt until decomposition. All polymers showed high elastic moduli of more than 4.1 GPa and tensile strengths of more than 52.6 MPa. In particular, the polymers obtained from TFMB, 2,2-bis(4-aminophenyl)hexafluoropropane (6FPD), and bis(4-aminophenyl)sulfone (pAPS) showed considerably high elastic moduli of 8.15, 5.07, and 5.26 GPa, respectively, and high tensile strengths of 118.9, 83.0, and 52.6 MPa, respectively. Through ultraviolet–visible (UV–Vis) spectroscopic analysis, at 420 nm, the TFMB-, 6FPD-, and pAPS-based polymers had high transmittances of 95.1%, 96.2%, and 91.2%, respectively, and low yellow indices of 2.37, 1.91, and 4.34, respectively, to produce mechanically robust, colorless, and transparent aramid films. Meanwhile, the polymer films obtained from the other diamines appeared slightly yellow. The details are described below.

## 2. Materials and Methods

### 2.1. Materials

TFMB was purchased from ITCHEM (Cheong-ju, Republic of Korea); a, a′-bis(4-aminophenyl)-1,4-diisopropylbenzene (APIPB), 2,2-bis[4-(4-aminophenoxy)phenyl]hexafluoropropane (APPFP), 3,4′-diaminodiphenylether (3,4-ODA), 6FPD, pAPS, 2,2′,5,5′-tetrachlorobenzidine (TCB), 4,4′-diaminodiphenylmethane (MDA), 4,4′-methylenebis(cyclohexylamine) (MBCHA), 4,4′-diaminodiphenylether (ODA), TPC, and IPC were purchased from TCI (Tokyo, Japan); 4,4′-methylenebis(2-chloroaniline) (MOCA), DMAc (anhydrous grade), and PO were purchased from Sigma-Aldrich (St. Louis, MO, USA). All reagents were used as received.

### 2.2. Synthesis of Aramids

Aramid polymers were synthesized in a 250 mL three-necked flask equipped with a reflux condenser, three-way stopcock, and magnetic bar. DMAc and diamine (10 mmol) were added to the flask and stirred until all amines were dissolved. The flask was then immersed in an ice bath and cooled to 0 °C. IPC (3 mmol) was added to the flask and stirred for 10 min, and then TPC (7 mmol) was added and stirred for 2 h. Afterward, PO was added to remove the HCl generated as a byproduct, and varnish was obtained.

### 2.3. Film Preparation

After casting the varnish on a glass substrate, it was dried in a convection oven at 40 °C for 30 min and 200 °C for 1 h to produce polymer films with thicknesses of several tens of micrometers.

### 2.4. Measurements

The viscosities of the varnishes were measured at 25 °C at a speed of 150 rpm using a DV2T viscometer (Brookfield, Waukesha County, WI, USA) equipped with a cone plate spindle CPA-52Z. FTIR absorption spectroscopy was performed in ATR mode using an FTIR-4100 spectrophotometer (Jasco, Tokyo, Japan). The ^1^H-NMR analysis was performed using an AVANCE III 500 NMR spectrometer (Bruker, Billerica, MA, USA) with DMSO-d_6_ as the solvent. DSC was performed using a Q2000 DSC (TA Instruments, New Castle, DE, USA) under a nitrogen atmosphere in a temperature range of 25–350 °C at a heating/cooling rate of 10 °C/min. TGA was performed using a Discovery SDT 650 TGA (TA Instruments) under a nitrogen atmosphere in a temperature range of 25–900 °C at a heating rate of 10 °C/min. Elastic moduli and tensile strengths were measured using UTM equipment (Yeonjin S-tech, Seoul, Republic of Korea) at a tensile rate of 1 mm/s. Visible-light transmittance and yellow index were measured using a V-650 UV–Vis spectrophotometer (Jasco, Japan). Dynamic thermomechanical analysis (DTMA) was performed using a Discovery DMA 850 (TA Instruments) under a nitrogen atmosphere in a temperature range of 25–380 °C at a heating rate of 5 °C min and a frequency of 1 Hz. X-ray diffraction (XRD) was performed using an X-ray Diffractometer (Panalytical Empyrean, Malvern, UK). Measurement was conducted using Cu Kα (1.54 Å) radiation operation at 40 kV and 30 mA.

## 3. Results and Discussion

### 3.1. Synthesis of Aramids and Basicity Effect of Diamine Monomers

Diamine monomers serve as nucleophiles in a nucleophilic acyl substitution reaction with diacid chloride to produce polyamides along with an acid byproduct, HCl, through the low-temperature solution condensation reaction [11]. However, diamine monomers may also act as bases, so they react with the acid byproduct to form an amine salt insoluble in organic solvents, resulting in loss of reactivity and gelation. Therefore, in the low-temperature solution condensation reaction, the basicity of diamine is a crucial factor for efficient polymerization. For TFMB, a high-viscosity transparent varnish was obtained without salt formation, [9,10] attributable to TFMB having a relatively low basicity due to the inductive effect of the strong electron-withdrawing CF_3_ group. In this study, for comparison with TFMB, various diamine monomers (TCB, pAPS, TFMB, MOCA, 6FPD, 3,4-ODA, APPFP, ODA, APIPB, MDA, MBCHA in Table 1) were examined for the low-temperature solution condensation reaction. Among them, TCB, pAPS, TFMB, MOCA, 6FPD, 3,4-ODA, and APPFP have electron-withdrawing groups (EWG), such as CF_3_, sulfone, and atomic halogen. As expected, they have relatively low basicity (pKa < 4.80) compared with other monomers because of the inductive effect of strongly withdrawing electrons from the functional amine group. Exceptionally, the relatively low basicity of 3,4-ODA despite the absence of EWG probably results from its asymmetric structure. Meanwhile, ODA, APIPB, MDA, and MBCHA with no EWG have higher basicity (pKa > 5.20). In particular, MBCHA (an aliphatic diamine) has the highest basicity (pKa 10.97) due to the lone pair localization in the amine group. The pKa values of these diamine monomers are listed in Table 1 [12]. As is known from a comparison between PPTA (a para-type aramid) and Nomex (a meta-type aramid), the diacid chloride linkage structure may also affect the reactivity and solubility during polymerization [13,14]. In this study, this effect was neglected to examine only the basicity effect of diamine monomers. Thus, diacid chloride was used with a mixture of TPC and IPC at a fixed molar ratio of 70:30 (Figure 1).

For the seven diamine monomers with lower basicity, an amine salt was not formed when diacid chloride was added to the solution, and the viscosity increased while the reaction system remained transparent (Case 1 in Figure 1). Notably, the rate of viscosity increase differed for the various monomers. The TFMB solution came close to gelation within approximately 10 min after adding the diacid chloride, showing the fastest increase in viscosity, attributable to the high rigidity of the growing polymer chain. Meanwhile, the other six monomers showed a gradual increase in viscosity. Among them, the rate of increase in viscosity for TCB was noticeably slow, so the viscosity increased to reach equilibrium over several hours, attributable to its very low nucleophilicity. The four atomic chlorine groups likely caused a strong inductive effect, significantly lowering the nucleophilicity of the monomer. All seven monomers could produce varnishes and films using the same method as that reported for TFMB in the literature (Case 1 in Figure 1). After adding diacid chloride to the diamine monomer solutions, when the reaction system reached an appropriate viscosity, PO was added to remove HCl, and the reaction solution was used as a varnish without purification to obtain films with a superb appearance and several tens of micrometer thickness. Consequently, diamine monomers with relatively low basicity could easily and conveniently provide aramid films through the one-pot reaction with diacid chloride in the same vessel. In solution condensation polymerization, by using an aprotic polar amide solvent such as DMAc or NMP and adding salts such as LiCl and CaCl_2_, the solubility of the polymer can be increased and thus a high molecular weight polymer can be obtained. Cosimbescu et al. produced a high-viscosity solution using CaCl_2_ as an additive in the process of manufacturing aramid using terephthaloyl dichloride and para-phenylenediamine as monomers and pyridine as an acid scavenger [15]. However, since such inorganic salts become impurities in the final product especially for the production of films, they must be removed by washing. In contrast, the present manufacturing method of converting the acid byproduct into volatile substances using PO has the advantage of allowing the polymer solution to be used as is without additional washing. Meanwhile, when the other aromatic diamine monomers, ODA and MDA, which have relatively higher basicity, were reacted in the same manner, amine salts were formed immediately after the addition of diacid chloride, making the reaction system opaque and insoluble. As expected, when these reaction solutions were poured into a mixed solvent of water and alcohol, almost no polymer was obtained. The cycloaliphatic diamine MBCHA also showed a similar result. This is because these highly basic monomers formed salts through an acid–base reaction with the acid byproduct during polymerization and lost their reactivity (Case 2 in Figure 1). Once the amine salt was formed, it did not dissolve again, even if more solvent was added or the temperature was increased. Notably, APIPB provided a fairly high-viscosity reaction solution without amine salt formation, despite having almost the same basicity as MDA. Therefore, APIPB belongs to the Case 1 monomers in this study. The reason is thought to be as follows. Unlike MDA with one methylene linkage (two Csp^2^–Csp^3^ single bonds), APIPB has two methylene linkages (four Csp^2^–Csp^3^ single bonds), which could have increased the main chain flexibility. As a result, the amine ends of the growing polymer chain were probably given great mobility, and the condensation reaction proceeded quickly enough to surpass the salt formation reaction with HCl. This result suggests that not only the diamine monomer basicity but also the growing polymer chain flexibility can greatly affect reactivity. According to the literature, although ODA has a fairly high basicity, when reacted with IPC alone, a solution with a fairly high viscosity is obtained without salt formation, from which a polymer film can be easily obtained, but it appears quite yellow [16]. However, because the polymer chain flexibility and coloring do not meet the purpose of this study, aramid film manufacturing using IPC alone was not examined.

**Figure 2:**
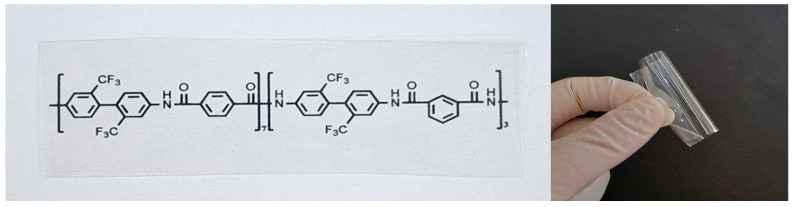
Aramid film (Run 3 in Table 1) manufactured from TFMB.

We examined the solubility of the polymers in several solvents and summarized the results in Table 2. Most polymers dissolved well in aprotic polar solvents such as DMAc, NMP and DMSO with solubility parameters (δ) of 22–27 MPa^1/2^. On one hand, although some polymers were partially soluble in acetone and THF, which have δ of 19–20 Mpa^1/2^, but most polymers were insoluble. On the other hand, all the polymers were completely insoluble in chloroform and toluene, which are non-polar solvents with δ of 19 Mpa^1/2^ or less, and was also completely insoluble in methanol, a very highly protic polar solvent with δ of 29.7 Mpa^1/2^. Because the obtained polymers were almost insoluble in THF, gel permeation chromatography analysis was impossible using this solvent as the eluent. Instead, the molecular weight and chain rigidity were inferred by measuring the viscosity of the polymer solutions in DMAc. The varnishes used in film production showed a high viscosity in the range of 160–2520 cP, even at a low concentration of 12 wt% (Table 1). Particularly, polymers from TFMB, MOCA, and APPFP had an extremely high viscosity over 1000 cP. This indicates that the polymers have a high molecular weight and/or chain rigidity. Because of the high viscosity, casting on a glass substrate was very easy, and thick films with a thickness of approximately 50–60 μm were produced through a typical convection drying process. The films had a smooth surface and a superb appearance and did not tear or break when pulled or bent by hand. Figure 2 shows images of the film manufactured from TFMB.

### 3.2. Characterization of Aramids

**Figure 3:**
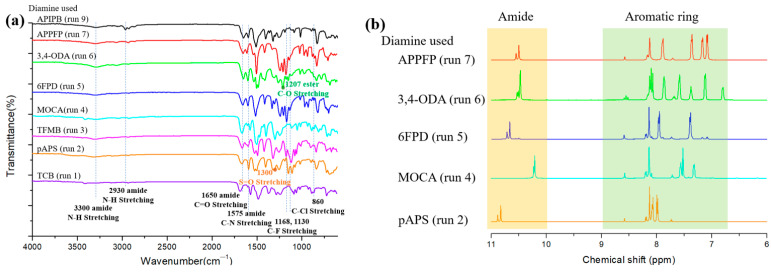
(**a**) FTIR (in ATR mode) and (**b**) ^1^H-NMR (in DMSO-d_6_) spectra of aramids prepared from different diamines. The run numbers in parentheses are from Table 1.

FTIR analysis of the polymers was performed (Figure 3a). For the fabricated polymers, absorption peaks were observed at ~1650 and 1575 cm^−1^, attributable to the amide C=O and C–N stretching, respectively. A broad absorption peak due to amide N–H stretching was observed within 3500–3100 cm^−1^, suggesting that the amide groups strongly interacted with each other through hydrogen bonding. In addition, absorption peaks due to specific functional groups (e.g., CF_3_ and sulfone) present in each monomer were observed. The ^1^H-NMR analysis was performed using DMSO-d_6_ as a solvent (Figure 3b). All polymers showed peaks due to aromatic protons around 7–9 ppm, and the peak due to amide protons shifted further downfield to appear at around 10–11 ppm. These results confirm that the aramid polymers were successfully synthesized as desired. 

### 3.3. Thermal Properties of Aramids

**Figure 4:**
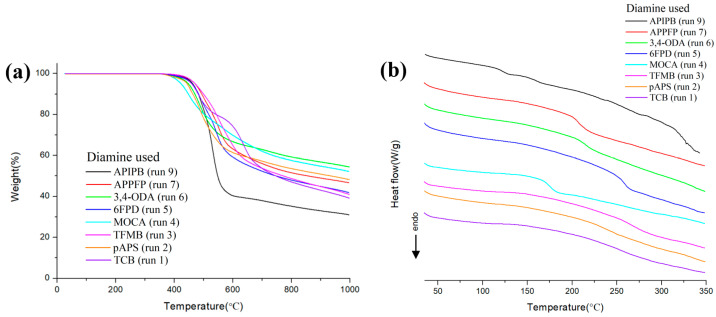
(**a**) TGA and (**b**) DSC curves of aramids prepared from different diamines (under N2 flow, a heating rate of 10 °C/min). The run numbers in parentheses are from Table 1.

The thermal decomposition stability of the polymers was evaluated using TGA (TGA curves in Figure 4a, Table 1). The thermal decomposition temperature (T_d5_) at a weight loss of 5 wt% under a nitrogen atmosphere was greater than 400 °C for all polymers, indicating that the polymers have very high thermal decomposition resistance. This thermal stability is attributable to the coplanar resonance structure comprising aromatic and amide groups in main chain [17,18]. DSC analysis was also performed to evaluate the thermodynamic stability of the polymers (Figure 4b). The polymers did not show any endothermic peak due to enthalpy change over a wide temperature range from room temperature to 350 °C, indicating that they do not melt even when the temperature is increased to near the decomposition temperature. Moreover, the TFMB-, pAPS-, and TCB-based polymers did not show any significant phase change due to glass transition in DSC curves. Therefore, DTMA was also examined although it often gives different results than DSC (Figure S1). DTMA was performed on the two optically important polymers made from TFMB and pAPS, which will be described later, and the two polymers showed glass transition at very high temperatures of 339 and 352 °C, respectively, as expected from the DSC results (Table 1). This is attributable the polymers having a rigid rod-like chain structure and strong hydrogen bonds between the chains, thereby requiring a very low entropy change and a high enthalpy change for the phase transition. For the APIPB-, APPFP-, 3,4-ODA-, 6FPD-, and MOCA-based polymers, which are thought to be relatively flexible, peaks due to glass transition were clearly observed around 121 °C, 207 °C, 214 °C, 257 °C, and 176 °C, respectively. 

### 3.4. Mechanical Properties of Aramids

**Figure 5:**
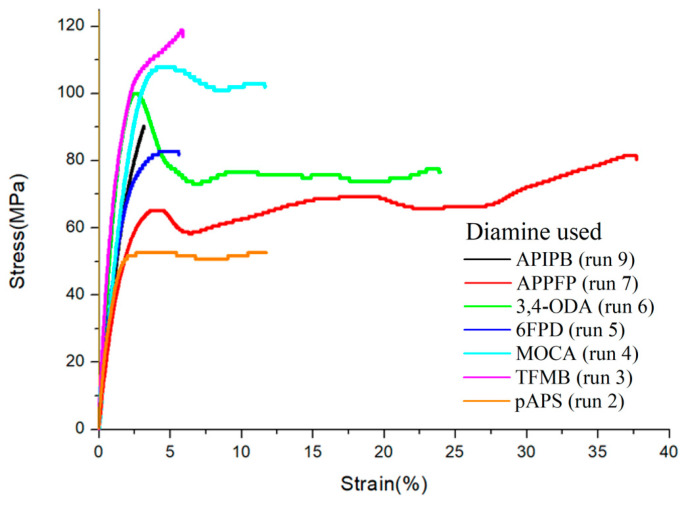
Stress–strain curves of aramid films prepared from different diamines. The run numbers in parentheses are from Table 1.

The stress–strain curves were analyzed using UTM (Figure 5, Table 1). All polymer films exhibited an elastic modulus of more than 4.1 GPa and a tensile strength of more than 52.6 MPa. Among them, the TFMB-based film had the highest elastic modulus and tensile strength of 8.2 GPa and 119 MPa, respectively, indicating a very rigid chain. The pAPS- and 6FPD-based films exhibited relatively high elastic moduli of 5.26 and 5.07 GPa, respectively, and high tensile strengths of 52.6 and 83.0 MPa, respectively. Meanwhile, the APPFP-based film had a lower elastic modulus and tensile strength of 4.1 GPa and 81 MPa, respectively, and the highest value of elongation at break (38%), indicating a less rigid chain. These values are comparable to those of the colorless and transparent polyimide and polyamideimide films reported so far [19,20,21]. For example, fluorinated polyimide obtained from TFMB and 4,4′-(hexafluoroisopropylidene)diphthalic anhydride (6FDA) has an elastic modulus of 3.1 GPa and a tensile strength of 120 MPa. The polyamideimide film synthesized by reacting 0.4-eq 6FDA and subsequently 0.6-eq TPC with TFMB has an elastic modulus of 5.1 GPa and a tensile strength of 110 MPa. 

### 3.5. Optical Properties of Aramids

**Figure 6:**
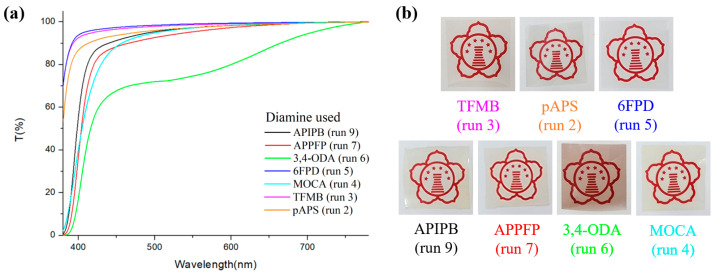
(**a**) UV–vis spectra and (**b**) images of aramid films made from different diamines. The run numbers in parentheses are from Table 1.

In fluorinated polyimides, a strong EWG, such as CF_3_, effectively suppresses intramolecular charge transfer to prevent yellowing [22,23]. This was true for all aramid films obtained in this study. The TFMB-based film had a high transmittance of 95.1% at 420 nm and a low yellow index of 2.4 (Figure 6, Table 1). The 6FPD-based film is optically superior with a slightly higher transmittance (96.2%) and lower yellow index (1.91). The pAPS-based film is also quite colorless and transparent; the transmittance and yellow index were 91.3% and 4.3, respectively. Although the pAPS-based film is somewhat inferior to the TFMB- and 6FPD-based films in terms of optical performance, these three films are colorless and transparent enough to be used as a transparent substrate or cover window for electronic devices. XRD analysis was conducted on these three films with superior optical properties (Figure S2). All the polymers exhibited a halo peak in wide angle region, indicating an existence in amorphous phase. Aramid films obtained from diamines other than TFMB, 6FPD, and pAPS appeared slightly yellow and had relatively low light transmittance in the visible region. For the MOCA-based film, the optical transmittance was 71.9% and the yellow index was 10.9. this chlorinated aromatic polymer is vulnerable to attack by radicals or anions because of the relatively low dissociation energy of the Csp^2^–Cl bond, so they probably formed unexpected color species during polymerization or convection drying. Similarly, the 3,4-ODA-based film showed the lowest light transmittance (54.5%) and the highest yellow index (17.0).

## 4. Conclusions

In this study, aramid polymers were synthesized via a low-temperature solution condensation reaction with diacid chloride using various diamine monomers. Diamines with relatively low basicity were smoothly polymerized without amine salt formation, resulting in highly viscous solutions. Meanwhile, more basic diamines generated amine salts during polymerization, rendering the reaction system opaque and failing to produce polymers. The acid byproduct generated during the polymerization process was removed without purification by converting it into an inert, highly volatile substance by adding PO. The obtained high-viscosity solutions were used as varnishes to obtain polymer films with excellent appearance. The films did not tear or break easily. In addition, the polymers did not easily decompose or melt, even when heated to 400 °C. For some polymers, glass transition was not observed until decomposition. The polymer films had high elastic modulus and tensile strength and were comparable to colorless transparent polyimide or polyamideimide films reported so far. Polymer films obtained from TFMB, 6FPD, and pAPS were particularly colorless and transparent. The synthesized polymer films are expected to be used as transparent substrates and cover windows for rollable and foldable display devices [24]. The method for removing acid byproducts by adding PO to the reaction system was very useful for easily and conveniently producing aramid films without a purification process. This study provides guidelines for the synthesis of colorless and transparent aramid films.

## Figures and Tables

**Table 1:** pKa values of diamine monomers and properties of aramids.

				Polymer Properties								
Run Number	Polymer	Salt Formation ^1^	PKa	Viscosity (cP)	Thickness (μm)	T_d5%_ (°C)	T_g_^2^ (°C)	Young’s Modulus (GPa)	Tensile Strength (MPa)	Elongation at Break (%)	T% at 420 nm	Y.I
1	TCB	X	1.58	160	NM	459	ND ^3^	NM ^4^	NM	NM	NM	NM
2	pAPS	X	2.41	280	50	443	352 ^5^	5.26	52.60	11.78	91.28	4.34
3	TFMB	X	3.23	2520	50	470	339 ^5^	8.15	118.90	5.93	95.13	2.37
4	MOCA	X	3.33	1060	60	424	176	4.95	107.84	11.58	71.87	10.94
5	6FPD	X	3.98	340	60	461	257	5.07	82.97	5.62	96.24	1.91
6	3,4-ODA	X	4.78	350	50	447	214	7.58	99.98	23.96	54.51	17.03
7	APPFP	X	4.80	1810	60	472	207	4.07	81.38	37.68	79.34	9.56
8	ODA	O	5.20	-	-	-	-	-	-	-	-	-
9	APIPB	X	5.31	820	60	463	121	4.76	89.60	3.14	85.50	7.16
10	MDA	O	5.32	-	-	-	-	-	-	-	-	-
11	MBCHA	O	10.97	-	-	-	-	-	-	-	-	-

^1^ O: salt formation, X: no salt formation; ^2^ measured by DSC unless otherwise noted; ^3^ ND: not detected; ^4^ NM: not measured; ^5^ measured by DTMA.

**Table 2:** Solubility of aramids manufactured in this study ^1^.

Solvent	Aramids, Diamine Used (Run Number in Table 1)							
(δ, Mpa^1/2^) ^2^	TCB (Run 1)	pAPS (Run 2)	TFMB (Run 3)	MOCA (Run 4)	6FPD (Run 5)	3,4-ODA (Run 6)	APPFP (Run 7)	APIPB (Run 9)
DMAc (22.8)	+	+	+	+	+	+	+	+
NMP (23.0)	+	+	+	+	+	+	+	−
DMSO (26.7)	+	+	−	+	+	+	+	±
Acetone (20.0)	−	−	±	−	±	−	−	−
THF (19.4)	−	−	−	−	−	−	±	−
Chloroform (19.0)	−	−	−	−	−	−	−	−
Toluene (18.2)	−	−	−	−	−	−	−	−
Methanol (29.7)	−	−	−	−	−	−	−	−

^1^ Solubility was evaluated by stirring 10 mg of polymer in 2 mL of solvent at room temperature for 1 day: (+) soluble; (±) partially soluble; (−) insoluble. ^2^ Solubility parameter. Abbreviations: DMAc, N,N-dimethylacetamide; NMP, N-methyl-2-pyrrolidone; DMSO, dimethylsulfoxide; THF, tetrahydrofuran.

## Data Availability

Data are contained within the article.

## Supplementary Materials

The following supporting information can be downloaded at: https://www.mdpi.com/article/10.3390/polym16050575/s1, Figure S1: DTMA Curves of aramids prepared from diamines of TFMB and pAPS; Figure S2: XRD patterns of aramids prepared from diamines of 6FPD, TFMB and pAPS.
