# Supplementary material for: Preparation and Properties of Mechanically Robust, Colorless, and Transparent Aramid Films
Source: Polymers (Basel). 2024 Feb 20;16(5):575. doi: 10.3390/polym16050575 (PMC10935007; doi:10.3390/polym16050575)
Supplement: Supplementary file 1 [file polymers-16-00575-s001.zip › polymers-2824030-supplementary.pdf]

Supplementary Material

Preparation and Properties of Mechanically Robust, Colorless, and Transparent Aramid Films

Heesang Kim [1], Jin-Hee Noh [1,2], Young-Rae Kim [1], Hyojin Kim [2,*] and Giseop Kwak [1,*]

[1] Polymeric Nanomaterials Laboratory, Department of Polymer Science & Engineering, Kyungpook National University, 1370 Sankyuk-Dong, Buk-Ku, Daegu 41566, Republic of Korea; wsk1025@naver.com (H.K.); jhnoh@dgtp.or.kr (J.-H.N.); xornjs147@naver.com (Y.-R.K.)

[2] Advanced Materials & Components Center, Industry Innovation Division, Daegu Technopark, 46-17 Seongseogongdan-ro, Dalseo-gu, Daegu 42716, Republic of Korea

* Correspondence: hjkim@dgtp.or.kr (H.K.); gkwak@knu.ac.kr (G.K.)

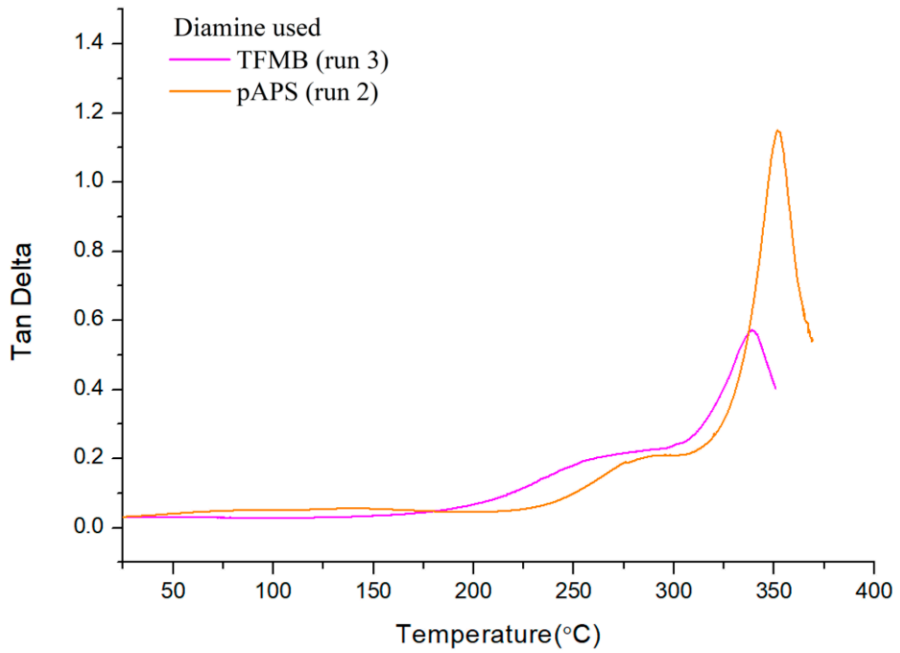

Figure S1. DTMA curves of aramids prepared from diamines of TFMB and pAPS (under N_2 flow, a heating rate of 5°C/min).

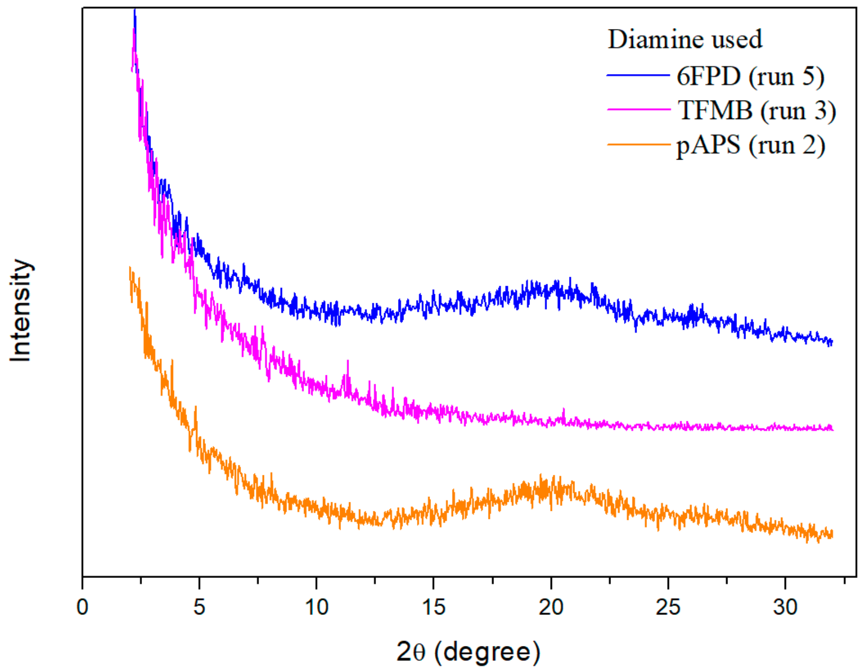

Figure S2. XRD patterns of aramids prepared from diamines of 6FPD, TFMB and pAPS.
